# Second-generation tau PET tracer in situ binding assessed with fluorescence imaging

**DOI:** 10.21203/rs.3.rs-11153643/v1

**Published:** 2026-10-27

**Authors:** Aleksandra Antic, Ambra Dreos, Tammaryn Lashley, Stephen Yong Yo-Ap, Kerstin Sander, Michael Schöll, Jörg Hanrieder

**Affiliations:** University of Gothenburg; University of Gothenburg; University College London; University College London; University College London; University of Gothenburg; University of Gothenburg

**Keywords:** Alzheimer’s disease, tau, PET tracer, GTP1, fluorescence microscopy

## Abstract

Brain positron emission tomography (PET) imaging is a valuable tool which allows visualization of the pathological structures inside the living patients’ brains. While amyloid-β PET tracers have been in use with Alzheimer’s disease patients in clinics, tau PET tracers have still not entered the clinical routine. Second-generation tau PET tracers have been developed with the aim to overcome the limitations of the first-generation tau PET tracers, such as low sensitivity and off-target binding. However, the detailed pathological characterization of these tracers’ binding is still needed for fully understanding their potential as accurate markers of pathogenic tau. Herein, we focused on the second-generation tau PET tracer Genentech Tau Probe 1 ([^18^F]GTP1) and made use of the fluorescent properties of its non-radioactive unlabeled variant GTP1 for correlative fluorescence microscopy. The results show that GTP1 binds to tau tangles and neuropil threads (NPTs), but not amyloid-β plaques. Importantly, our results indicate that there is a strong correlation between the fluorescent GTP1 signal and the [^18^F]GTP1 signal detected with autoradiography, which is currently the standard way of studying the tau PET tracer binding *in situ*. Together, this data shows the robust application of a non-radioactive tau PET tracer in binding studies in *post-mortem* tissue and sheds new light on the tau binding properties of GTP1.

## 1. INTRODUCTION

Despite Alzheimer’s disease (AD) being the most common cause of dementia, the details of pathological mechanisms underlying this disorder still remain unknown, significantly hampering the development of effective treatment strategies. AD is characterized by two major pathological hallmarks, with the first one being the progressive abnormal accumulation and deposition of amyloid-β (Aβ) peptides in the form of extracellular plaques. Besides Aβ plaques, neurofibrillary tangles composed of hyperphosphorylated microtubule-associated protein tau are also found in the patients’ brain tissue [6]. The most widely accepted hypothesis suggests that Aβ aggregation and plaque formation are critical early events in AD pathogenesis that initiate a cascade of detrimental downstream events such as synaptic changes, tau pathology and eventually neurodegeneration [10, 26]. As the definitive confirmation of AD is currently possible only after the pathological *post-mortem* examination, PET imaging is a crucial tool for evaluating the patients’ brain pathology *in vivo*. Even though fluid biomarkers have been showing a lot of potential in terms of diagnosing AD, it is only brain imaging that enables *in vivo* detection of pathological structures with the spatial dimension, allowing for allocation of protein aggregation to specific brain regions.

While amyloid-β PET tracers have been in use in clinics, tau PET tracers have still not entirely entered the clinical routine (only tau PET tracer [^18^F]flortaucipir has been approved for clinical use) [7, 22]. However, unlike amyloid-β, pathological tau load has been shown to correlate with cognitive decline and AD disease progression [1, 11]. Tau PET tracers have the potential to improve diagnostic accuracy, disease staging, patient selection for specific treatments and clinical trials for AD, as well as monitoring the disease progression and treatment effects [19, 25, 27, 31].

Second-generation tau PET tracers have been developed with the aim to overcome the limitations of the first-generation tau PET tracers, such as low sensitivity and off-target binding [4, 13, 29]. However, detailed characterization of these tracers’ binding is still needed for fully understanding their potential as accurate markers of pathogenic tau. *In situ* binding studies of the tracers typically require the use of autoradiography, which comes with limitations such as the need for radiolabeled tracers, low spatial resolution and the lack of possibility for integration with other chemical imaging modalities on the same sample.

Herein, we made use of fluorescent properties of the unlabeled (non-radioactive) tau tracer molecule Genentech Tau Probe 1 (GTP1) (Fig. 1a) and performed fluorescence microscopy-based assessment of its *in situ* binding. This enabled acquiring multichannel fluorescence imaging data and, consequently, GTP1 was imaged on the same tissue section as the anti-tau and anti-amyloid-β antibodies, as well as the fluorescent nuclear stain. Furthermore, investigation of the relationship between the GTP1 and the AT8 fluorescent signals across distinct pathological tau aggregates was examined. Finally, the correlation between GTP1 fluorescence data and [^18^F]GTP1 autoradiography data was shown for the first time.

## 2. MATERIALS AND METHODS

### 2.1 Material

Fresh frozen brain tissue samples were obtained from clinically and neuropathologically diagnosed AD patients (Table 1). Both frontal and temporal cortex were obtained from three patients, while only frontal cortex was obtained from two patients and only temporal cortex from six patients. All the cases originate from the brain donation program of the Queen Square Brain Bank for Neurological Disorders, Department of Clinical and Movement Neurosciences, UCL Queen Square Institute of Neurology. Standard diagnostic criteria were used for the neuropathological diagnosis of AD [6, 18, 28].

### 2.2 Quantitative phosphorimaging

The frozen tissue sections were thawed and air-dried, then subsequently fixed in methanol (20 min). This was followed by rehydration in tris-buffered saline (30 min) and incubation in [^18^F]GTP1 solution (2 MBq/ml in tris-buffered saline) (15 min). The sections were finally washed in water (5 min) and 50% ethanol (5 min).

The serial dilution of the [18F]GTP1 solution which was used for the incubation of the brain sections acted as internal standards. These were absorbed onto filter paper, and were air-dried, along with the brain sections. This was followed by overnight exposure to the phosphor screen (BAS-IP MS, GE Healthcare). Typhoon Trio scanner (GE Healthcare) was used for phosphorimaging at the spatial resolution of 50 μm.

Phosphor images were analysed with ImageJ (version 1.48). Internal standards were used to generate correlation curves, based on which the quantification of [^18^F]GTP1 binding.

### 2.3 Immunohistochemistry

12-μm-thick human brain tissue sections were collected at −20°C inside a cryostat (Leica CM1950, Leica Biosystems) from fresh frozen tissue blocks kept in −80°C conditions prior to the sectioning. The optimal temperature component (OCT) (VWR chemicals, Avantor, Cat. #361603E) was used for embedding the tissue during the sectioning process and the sections were collected on microscopy glass slides (Epredia, Cat. # J1800AMNZ).

Importantly, the frontal cortex tissue sections used in the histochemical experiments aiming to relate the tau pathology load observed with fluorescence and that observed by autoradiography, were adjacent and mirrored to the sections used in autoradiography experiments.

Histochemical experiments started with a 10-minute-long drying of the tissue sections in vacuum inside a desiccator. Then, the sections were incubated in the gradient concentration of solutions: 95% ice cold ethanol (10 min), 70% ice cold ethanol (1 min) and 1xPBS (10 min). This was followed by incubation with blocking solution (2% Bovine Serum Albumin [BSA], 10% Normal Goat Serum [NGS], 0.1% Triton X-100, 0.07% TWEEN 20 in PBS) for a period of 90 min at room temperature.

The samples were then incubated overnight at 4°C in the primary antibody solution consisting of a primary antibody and antibody diluent (0.1% NGS, 0.2% TWEEN 20 in PBS). The primary antibodies used were AT8 (0.2 mg/ml; Invitrogen, Thermo Fisher Scientific; Cat. # MN1020) and anti-β-amyloid (1–16) antibody (1 mg/ml, Biolegend, Cat. # 803003). Both AT8 and anti-β-amyloid antibodies were diluted 200x with the antibody diluent.

The following day the sections were washed in 0.1% PBST (PBS with 0.1% TWEEN 20) (3 x 5 min) and incubated for 1 h with the secondary antibody solution consisting of a secondary antibody and antibody diluent. This, and all the following steps, were performed in the dark. The secondary antibody used was Alexa Fluor 568 (2 mg/ml, Invitrogen, Thermo Fisher Scientific; Cat. # A11031). Alexa Fluor 647 (Invitrogen, Thermo Fisher Scientific, Cat. # A32728) was used only in combination with the anti-β-amyloid (1–16) primary antibody, in the plaque staining experiments. Both secondary antibodies were diluted 1000x in the antibody diluent. After the incubation, the tissue sections were washed in PBS (3 x 5 min).

Then, the samples were incubated for 30 s in a TrueBlack lipofuscin autofluorescence quencher solution (Biotium, Cat. # 357-23007) (5% TrueBlack solution in 70% ethanol) and subsequently washed in PBS (3 x 5 min). This was followed by a two-hour-long incubation with 100 μM GTP1 working solution (aq. solution with 5% DMSO). The working solution was made starting from a 2 mM GTP1 stock solution (in DMSO), sonicated for 1 h and then diluted in water. After the incubation with the working GTP1 solution, the sections were washed in PBS (3 x 5 min) and incubated for 15 min with the nuclear dye TO-PRO-3 iodide solution (Invitrogen, Thermo Fisher Scientific, Cat. # T3605), 1000x diluted in PBS. The tissue sections were then washed in PBS (3 x 5 min) and finally the antifade mountant ProLong^™^ Gold antifade (Invitrogen, Thermo Fisher Scientific, Cat.# P36934) and the coverslips (Menzel Gläser, 24x60 mm; Epredia) were added. The samples were imaged the following day.

The water used in the experiments was always deionized water obtained by the Milli-Q purification system (Millipore Corporation, Merck, Darmstadt, Germany).

### 2.4 Fluorescence microscopy

The chemically stained human brain sections were imaged with the widefield microscopy system SLIDEVIEW VS200 research slide scanner (Olympus), in the fluorescence mode with the Penta “Sedat” DAPI/FITC/Cy3/Cy5/Cy7 LED HC Filter Set and the UPlanXApo 20x/0.8 objective. The tiled images of entire tissue sections were obtained in three channels (DAPI, Cy3 and Cy5). The software used was VisiView (Visitron Systems GmbH).

For acquiring hyperspectral data, the Carl Zeiss LSM980 confocal microscopy system with a 32-channel GaAsp detector was used in combination with the ZEN Blue imaging software (Carl Zeiss Microscopy GmbH). Excitation was performed with the 405 nm laser line and the objective used was Plan-Apochromat 20x/0.8.

### 2.5 Fluorescence image analysis

For image analysis, QuPath v0.5.1 [2] and Fiji (ImageJ v1.54p) [24] were used. Images of the tissue sections containing three fluorescence channels were first imported in QuPath. Fields of view (FOVs) (~ 62540 μm^2^) within grey matter were selected and then exported from QuPath to Fiji.

In Fiji, three channels were first separated into individual images. Binary masks were applied to the DAPI channel image (GTP1 staining) and the Cy3 channel image (AT8 staining). The binary images generated were then used to measure the total area positive for each of the dyes. For tangles analysis, each of the FOVs was inspected individually - both as a fluorescent image and a binary image - and tangles were manually selected based on the shape and size of the structures. For threads analysis, criteria for automatic selection of particles were applied to the binary images - circularity: 0–0.7, and area: 2–75 μm. The resulting ROIs were reviewed manually and if a particle had been recognized as a part of a tangle before, it was excluded from the list of threads ROIs.

The binary masks for the GTP1 channel image and the AT8 channel image were also combined into a single union binary image for each FOV, which was achieved with the Image Calculator function “Add image” in Fiji. The total area of a tangle was then considered to be the area of the union of the GTP1-positive ROI and the corresponding AT8-positive ROI. For each tangle, it was then calculated how much of its total area is captured by GTP1 only, AT8 only and by both GTP1 and AT8.

The quantitative data was based on the raw unaltered images. The images included in the article as representative were adjusted for easier visualization in terms of brightness and contrast (applied to the entire image equally).

### 2.6 Statistical analysis

Statistical analysis was performed in GraphPad Prism version 10.4.1 (GraphPad Software).

In all statistical tests p ≤ 0.05 was considered a statistically significant value.

The first analysis aims to describe the relationship between the tau pathology load observed with fluorescent GTP1 and that measured with [^18^F]GTP1 autoradiography. Tau pathology load measured with fluorescence is given as the area positive for GTP1 within total FOV area (%) and averaged across 5 FOVs per patient (Fig. 2a). The same approach was used for GTP1-positive tangles area and GTP1-positive threads area (Fig. 2d,e). The autoradiography data is based on an entire tissue section, adjacent and mirrored to the one used for the tau pathology load assessment with fluorescent GTP1. Linear correlation was assumed between the tau pathology loads assessed in these two ways and it was evaluated using Pearson correlation analysis.

Next, it was assessed how GTP1 performs as a fluorescent tau pathology stain in comparison to AT8. Total load positive for the dye, tau tangles load and threads load were each expressed as the area within the total FOV area and averaged across 10 FOVs per patient for frontal cortex and 5 FOVs per patient for temporal cortex. Paired two-tailed t-test was performed between the GTP1 and the AT8 group for frontal cortex and then separately between the GTP1 and the AT8 group for temporal cortex (Fig. 4).

Further, the data from the GTP1-AT8 union images was introduced so that it can be assessed how much of the area taken by the AT8-positive tangles is also GTP1-positive. Double positive AT8+/GTP1 + tangle area was expressed as a fraction within the area occupied by the AT8-positive tangles and averaged across 10 FOVs per patient for frontal cortex and 5 FOV per patient for temporal cortex (Fig. 5a). Similarly, it was assessed how much of the area taken by the GTP1-positive tangles is also AT8-positive, which was expressed as a fraction of double positive GTP1+/AT8 + tangle area within the area occupied by the GTP1-positive tangles (Fig. 5b)

The analysis was also done at the level of individual tangles to explore how much of the total area of a tangle is captured by GTP1 and how much by AT8. This was expressed as a fraction of an area covered by the stain within the total area of a single tangle. Visual representation is given in the form of graphs for all the frontal cortex tangles, temporal cortex tangles and both groups combined (Fig. S1). Further, these values were averaged across 10 FOVs per patient for frontal cortex and 5 FOVs per patient for temporal cortex. For the comparison between the GTP1 group and AT8 group, paired two-tailed t-test was performed (Fig. 6).

## 3. RESULTS

### 3.1 GTP1 emission is detectable in the UV/vis spectrum

Before investigating *in situ* fluorescent staining properties of GTP1, characterization of the spectral properties of GTP1 was performed. As the first step, the GTP1 properties *in vitro* in ethanol were assessed. Here, GTP1 emission at the excitation wavelength of 350 nm was detectable in the UV and the visible light spectrum with a peak at 480 nm (Fig. 1b).

Next, the GTP1 emission was assessed in the brain tissue section at the level of a tau tangle. The GTP1-labelled structures were detectable in the blue and the green channel during fluorescence microscopy. In order to establish the GTP1 emission spectrum in the brain tissue at the level of a tau tangle, imaging was performed with a confocal microscope equipped with a 32-channel spectral detector. This way, the signal intensity can be assessed for the short interval of 9 nm throughout the 400–600 nm range. The signal intensity from each of the 32 channels was used to construct an emission spectrum of GTP1 at the excitation wavelength of 405 nm (Fig. 1c).

### 3.2 GTP1-positive tangle load correlates with [^18^F]GTP1 autoradiography tau burden

The key aim was to determine if fluorescence microscopy and autoradiography yield comparable results in terms of tau pathology load assessed with GTP1. Firstly, the entire fluorescent GTP1-positive area was considered, without any stratification or consideration for the structure types. The analysis showed that GTP1-positive area in the fluorescence images correlates positively and very strongly with the [^18^F]GTP1 autoradiography results for frontal cortex (Fig. 2a) (Pearson coefficient = 0.885, p = 0.046, CI = [0.012, 0.099], R^2^ = 0.783).

The GTP1-positive structures were then stratified, and the majority of the GTP1-positive area was shown to consist of tangles and threads (Fig. 2b). On average, around 66% of GTP1-positive area in frontal cortex and 69% in temporal cortex is made up of tangles. Threads make up on average around 19% of GTP1-positive area in the frontal cortex and 15% in temporal cortex. Additionally, most of the GTP1-positive area consists of tangles and threads across all patients, both in case of frontal and temporal cortex (Fig. S2).

Further, rather than the entire fluorescent GTP1-positive area, the correlation between GTP1-positive area made up of tangles and threads and the autoradiography data was examined. The fluorescent GTP1-positive area made up of tangles and threads correlates positively and very strongly with the [^18^F]GTP1 autoradiography results for the frontal cortex (Fig. 2c) (Pearson correlation coefficient r = 0.934, p = 0.020, CI = [0.296, 0.100], R^2^ = 0.873).

Finally, the fluorescent GTP1-positive area made up of tangles only, correlates the strongest with the [^18^F]GTP1 autoradiography results for the tau pathology load in the frontal cortex (Fig. 2d) (Pearson correlation coefficient r = 0.979, p = 0.004, CI = [0.708, 0.999], R^2^ = 0.958).

### 3.3 GTP1 stains larger tau tangles area than AT8

The performance of GTP1 as a fluorescent stain that targets tau pathology was examined through co-staining with the already established anti-ptau antibody AT8. The staining with nuclear dye TO-PRO-3 iodide was also performed on the same samples (Fig. 3).

First, the analysis showed that the total area positive for the stain was larger in the case of GTP1 than it was in the case of AT8 staining (Fig. 4b). Paired two-tailed t-test confirmed that there was a significant difference between the GTP1 and AT8 group, in both frontal (5 patients, p = 0.044, CI = [−1.573, −0.034]) and temporal cortex (9 patients, p = 0.006, CI = [−0.545, −0.124]).

Additionally, the area made up of tangles was larger in the case of GTP1 staining than it was in the case of AT8 (Fig. 4c). With paired two-tailed t-test it was confirmed that there was a significant difference between the GTP1 and AT8 group in case of both frontal (5 patients, p = 0.010, CI = [−1.559, −0.382]) and temporal cortex (9 patients, p = 0.001, CI = [−0.545, −0.217]). However, the area occupied by threads did not differ significantly when the two stains were compared (Fig. 4d). Paired two-tailed t-test for frontal cortex (5 patients): p = 0.550, CI = [−1.233, −0.145]; for temporal cortex (9 patients): p = 0.061, CI = [−0.139, −0.004].

### 3.4 GTP1 stains AT8-positive tangles, but AT8 does not always stain GTP1-positive tangles

The confirmation that GTP1 stains a larger area made up of tangles compared to AT8, was followed by the exploration of whether all the AT8-positive tangles were detectable with the GTP1 staining. Therefore, an analysis was performed to answer how much of the AT8-positive area made up of tangles was captured by GTP1 staining and vice versa - how much of the GTP1-positive area made up of tangles was captured by AT8 staining. It was first observed that **GTP1 staining captures AT8-positive tangles** (Fig. 5a). In fact, the AT8-positive area occupied by tangles was almost entirely also GTP1-positive. On average, around 99% of the AT8-positive area covered by tangles in frontal cortex, and 98% in temporal cortex, was also GTP1-positive. On the other hand, **GTP1-positive tangles were not always captured by AT8 staining** (Fig. 6b). On average, only around 45% of GTP1-positive area covered by tangles in frontal cortex, and 65% in temporal cortex, was also AT8-positive.

### 3.5 GTP1 and AT8 label different areas within the same tangle

Next, the focus was moved towards only those tangles which were positive for both GTP1 and AT8 (double positive). Herein, the goal was to determine how the two stainings differed at the level of individual tangles. It was observed that GTP1 and AT8 bind to different areas within the same tangle (Fig. 6, Fig. S1). GTP1 stained on average around 74% of the area of a double positive tangle in frontal cortex, while AT8 stained on average around 54%. Similarly, in temporal cortex GTP1 labelled on average 74% of the area of a double positive tangle and AT8 labelled 60%. Two-tailed paired t-test confirmed that the difference between the GTP1 and AT8 group was significant, both in frontal cortex (5 patients, p = 0.022, CI = [−27.25, −11.72]) and temporal cortex (9 patients, p = 0.033, CI = [−24.86, −1.381]).

### 3.6 GTP1 does not bind to amyloid-β plaques

Finally, the staining of amyloid-β plaques, as the second major hallmark of AD, was considered. With anti-amyloid-β antibody (1–16) (6E10) co-staining, it was confirmed that GTP1 does not bind to amyloid-β plaques (Fig. S3).

## 4. DISCUSSION

Herein, a fluorescence microscopy strategy for investigating tau PET tracers binding *in situ* is presented. Due to the photochemical properties of the non-radioactively labelled second-generation tau PET tracer GTP1, it was possible to assess its binding to tau aggregates in this way.

[^18^F]GTP1 has been shown to have desirable PET tracer characteristics, such as high affinity and selectivity for AD tau pathology, good penetration in brain and rapid brain uptake and clearance [23]. However, a major challenge with tau PET imaging is the insufficient spatial resolution for accurate assessment of on- and off-target binding. *In situ* tracer binding is typically assessed with autoradiography, which requires synthesis and handling of radioactively labelled tracer molecules. Our approach overcomes these challenges, enabling both submicron spatial resolution and a more accessible experimental setup that does not require the use of radioactive chemicals. Additionally, the tissue samples labelled with fluorescent GTP1 allow for the use of correlative imaging modalities on the same sample.

Importantly, our results show that the presented GTP1 fluorescence microscopy method and [^18^F]GTP1 autoradiography yield comparable accuracy in terms of assessing tau pathology load in AD. Very strong positive correlation between the two in the case of total tau pathology load improves even further when only tau tangles are considered, indicating that it is primarily tau tangles that are being detected by [^18^F]GTP1.

Further, the GTP1 binding to tau tangles was validated by co-staining experiments with anti-ptau antibody AT8, which confirmed that GTP1 binds to the tau tangles captured by AT8. Qualitatively, the results show that the area stained by GTP1 and that stained by AT8 do not overlap entirely and that they, at times, label different parts of the same tangle. It was also observed that GTP1 often covers larger area of a tau tangle compared to AT8, which was further quantitatively confirmed. This is likely the result of GTP1 and AT8 binding to different epitopes within tau structures. Phosphorylation at tau Ser202 and Thr205 are recognized as necessary for AT8 binding [3, 8], but phosphorylation at tau Ser199 [3] and Ser208 [14, 21, 30] have also been implicated. On the other hand, GTP1 molecules bind inside a groove containing the amino acid sequence 351–360, formed by each of the two tau protofilaments within the paired helical filament (PHF) structures [15].

Interestingly, a larger number of tau tangles was detected by GTP1 than by AT8. This might indicate that GTP1 binds to a wider spectrum of tau tangle maturation stages in comparison to AT8, which is known to mostly bind to earlier tau tangle maturation stages [9, 16, 17]. Alternatively, GTP1 could be binding off-target to other components within cells resulting in a tangle-like fluorescent signals. However, it has previously been reported that [^18^F]GTP1 shows no apparent off-target binding including amyloid plaques [20, 23]. Therefore, further investigation is needed for a conclusive explanation regarding this issue.

Limitations of the study include the small number of cases included. This is especially relevant when it comes to the correlation between fluorescence and autoradiography data, since only five cases were available with matched data. Therefore, even though the presented data provides strong initial results, extended follow up studies with larger cohorts are needed. Furthermore, it was only frontal cortex that was included in the comparison between autoradiography and fluorescence imaging method for accessing tau pathology load. It would be beneficial to confirm the validity of this method in other brain regions, especially those involved in earlier Braak stages (transentorhinal and entorhinal region, temporal cortex) [5, 12]. Finally, the study included only AD cases. Even though there are indications that GTP1 binds exclusively to PHF in AD – at least when it comes to the binding mechanism described by Merz et al. [15] – including other tauopathies in this type of studies could help confirm the specificity of GTP1 and its diagnostic potential for AD.

## 5. CONCLUSION

Brain amyloid-β and tau PET tracers are crucial biomarker tools available to neuroscientists and clinical neurologists for establishing AD diagnosis *in vivo*. Additionally, tau PET also has a prognostic marker potential. Therefore, tau PET tracer development and characterization are of central interest in the diagnostic process and drug development. In this study, the accuracy of the fluorescence imaging approach for studying second-generation tau PET tracer GTP1 tau binding *in situ*, is confirmed through the comparison with the established [^18^F]GTP1 autoradiography method. We also show that GTP1 binds to both tau tangles and neuropil threads, and that, in the case of tangles, this binding is greater than AT8 binding, both in terms of the number of stained structures and the area stained within individual tangles.

## Supplementary Material

This is a list of supplementary files associated with this preprint. Click to download.

- Anticetal21006ActaSI.pdf

## Figures and Tables

**Figure 1:**
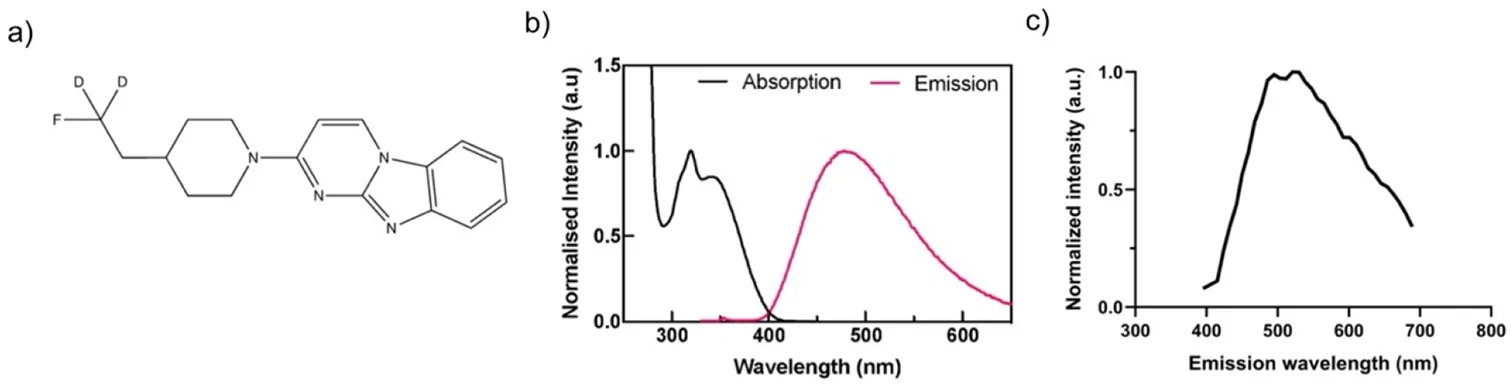
Spectroscopic characterization of GTP1 a) Chemical structure of non-labelled non-radioactive GTP1 molecule. b) Absorption and emission spectra of GTP1 in ethanol (excitation: 350 nm; Cary Eclipse spectrophotometer, Agilent). c) Emission profile of GTP1 in tissue at the level of a tau tangle (excitation: 405 nm).

**Figure 2:**
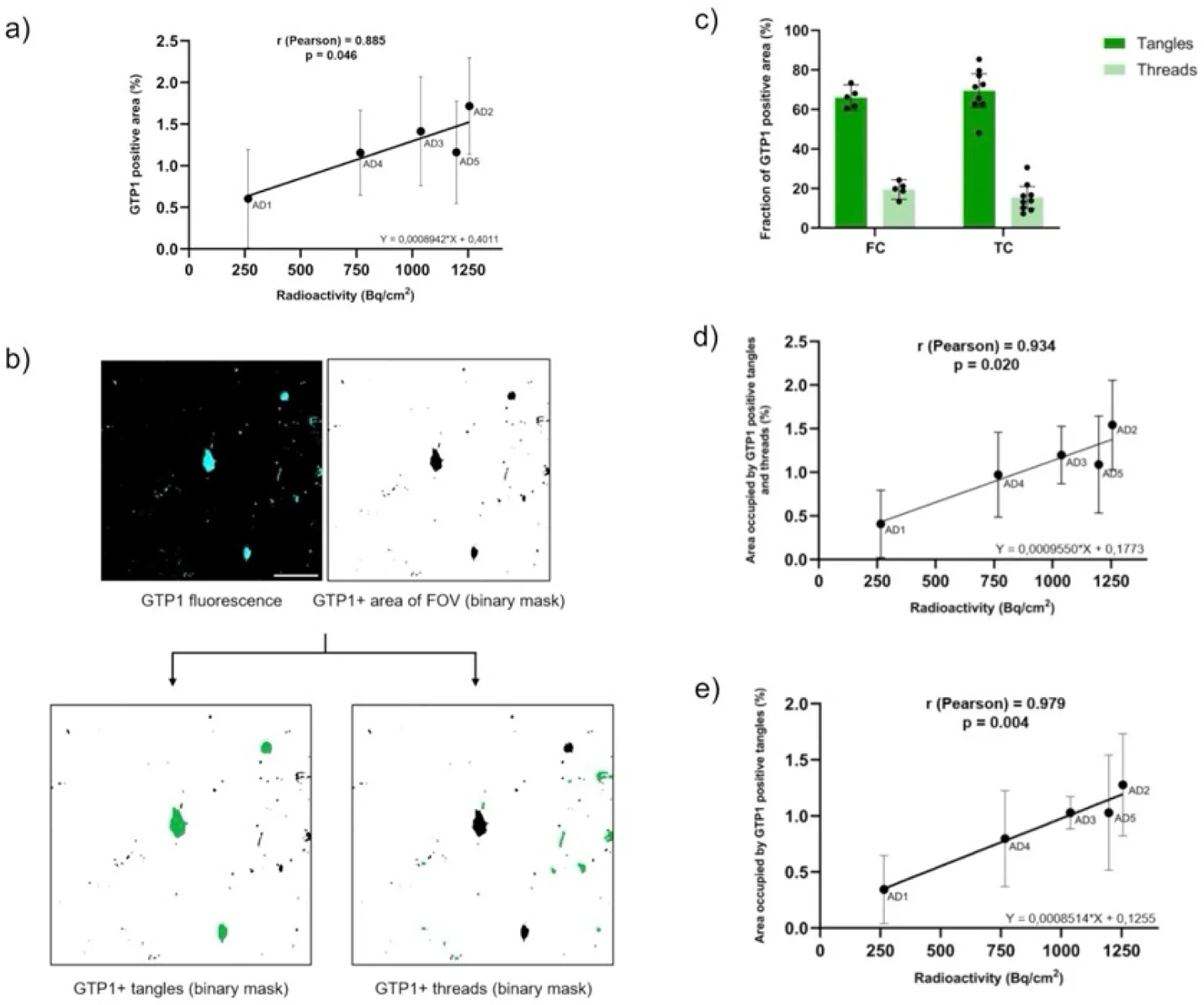
The area consisting of fluorescent GTP1-positive tangles correlates positively and very strongly with the [^18^F]GTP1 autoradiography tau burden data a) GTP1-positive area correlates positively and very strongly with the autoradiography results for frontal cortex. Pearson coefficient = 0.885, p = 0.046, CI = [0.012, 0.099], R^2^ = 0.783. The area positive for GTP1 is given as a fraction within the total area (%) and shown for each patient (5 cases, frontal cortex). b) The majority of the GTP1-positive area consists of tau tangles and threads. The shown values represent area fraction occupied by tangles or threads within GTP1-positive area, given as percentage for each case (5 cases for frontal cortex and 9 cases for temporal cortex). The error bars represent the 95% confidence interval. c) Tau pathology stratification method. Binary masks were generated from the FOV images of the GTP1 fluorescent signal. Tau tangles ROIs and tau threads ROIs were selected according to the criteria (described in the Methods section) and their area was measured. Scale bar: 50 μm d) The GTP1-positive area made up of both tangles and threads correlates positively and very strongly with the autoradiography results for frontal cortex. Pearson correlation coefficient r = 0.934, p = 0.020, CI = [0.296, 0.100], R^2^ = 0.873. The area occupied by both GTP1-positive tangles and threads is given as a fraction within the total area and shown for each patient (five patients, frontal cortex). e) **The correlation with autoradiography tau burden data is the strongest in the case of fluorescent GTP1-positive area made up of tangles only**. Pearson correlation coefficient r = 0.979, p = 0.004, CI = [0.708, 0.999], R^2^ = 0.958. The area occupied by GTP1-positive tangles is given as a fraction of the total area (%) and shown for each patient (5 patients, frontal cortex). a),c),d) Error bars represent standard deviation.

**Figure 3:**
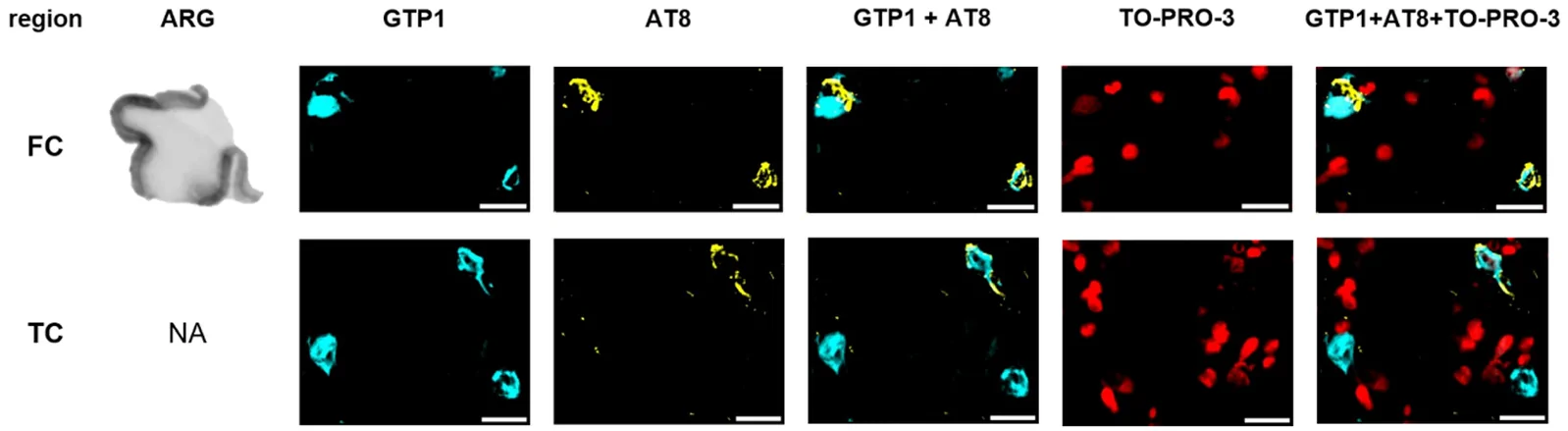
Binding profile of [^18^F]GTP1 detected by autoradiography and fluorescent staining patterns of GTP1 and AT8 (together with the nuclear stain TO-PRO-3 iodide) in Alzheimer’s disease brain samples Binding profile of [^18^F]GTP1 in the frontal cortex tissue section adjacent to the sections on which histochemistry and fluorescence imaging were performed - shown in grayscale. Fluorescent staining patterns of GTP1 (turquoise) and AT8 (yellow), as well as the nuclear stain TO-PRO-3 iodide (red) shown in the color images. Images from the AD3 case. FC - frontal cortex, TC - temporal cortex, ARG - autoradiography, NA - not available, scale bar: 20 μm.

**Figure 4:**
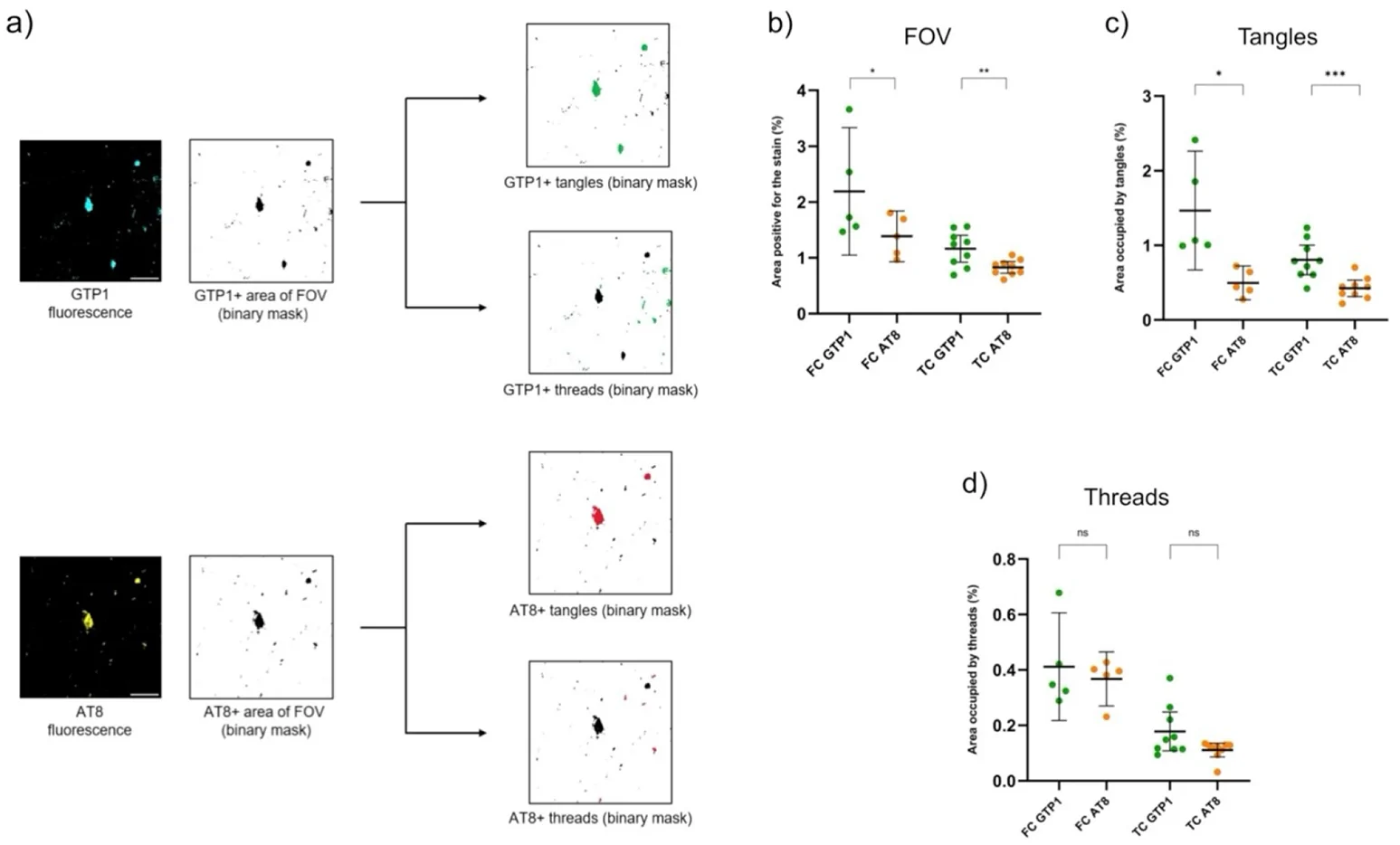
GTP1 stains a larger tangles area than AT8, but there is no difference in terms of neuropil thread-area stained a) Tau pathology stratification method. Binary masks were generated from the FOV images of the GTP1 fluorescent signal and of the AT8 signal. Tau tangles ROIs and tau threads ROIs were selected according to the criteria (described in the Methods section) and their areas were measured. Scale bar: 50 μm b) Paired t-test shows that area positive for the stain is significantly larger with GTP1 staining than with AT8 staining, both in case of FC (5 patients, p = 0.044, CI = [−1.573, −0.034]) and TC (9 patients, p = 0.006, CI = [−0.545, −0.124]). c) GTP1 stains larger tangle area than AT8 (FC, 5 patients, paired t-test: p = 0.010, CI = [−1.559, −0.382]; TC, 9 patients, paired t-test: p = 0.001, CI = [−0.545, −0.217]). d) The area occupied by threads does not differ significantly between the two stains (FC, 5 patients, paired t-test: p = 0.550, CI = [−1.233, −0.145]; TC, 9 patients, paired t-test: p = 0.061, CI = [−0.139, −0.004]). b) - d) The area is given as a fraction of the total area (%). Each data point corresponds to a single patient. The error bar shows 95% confidence interval (CI).

**Figure 5:**
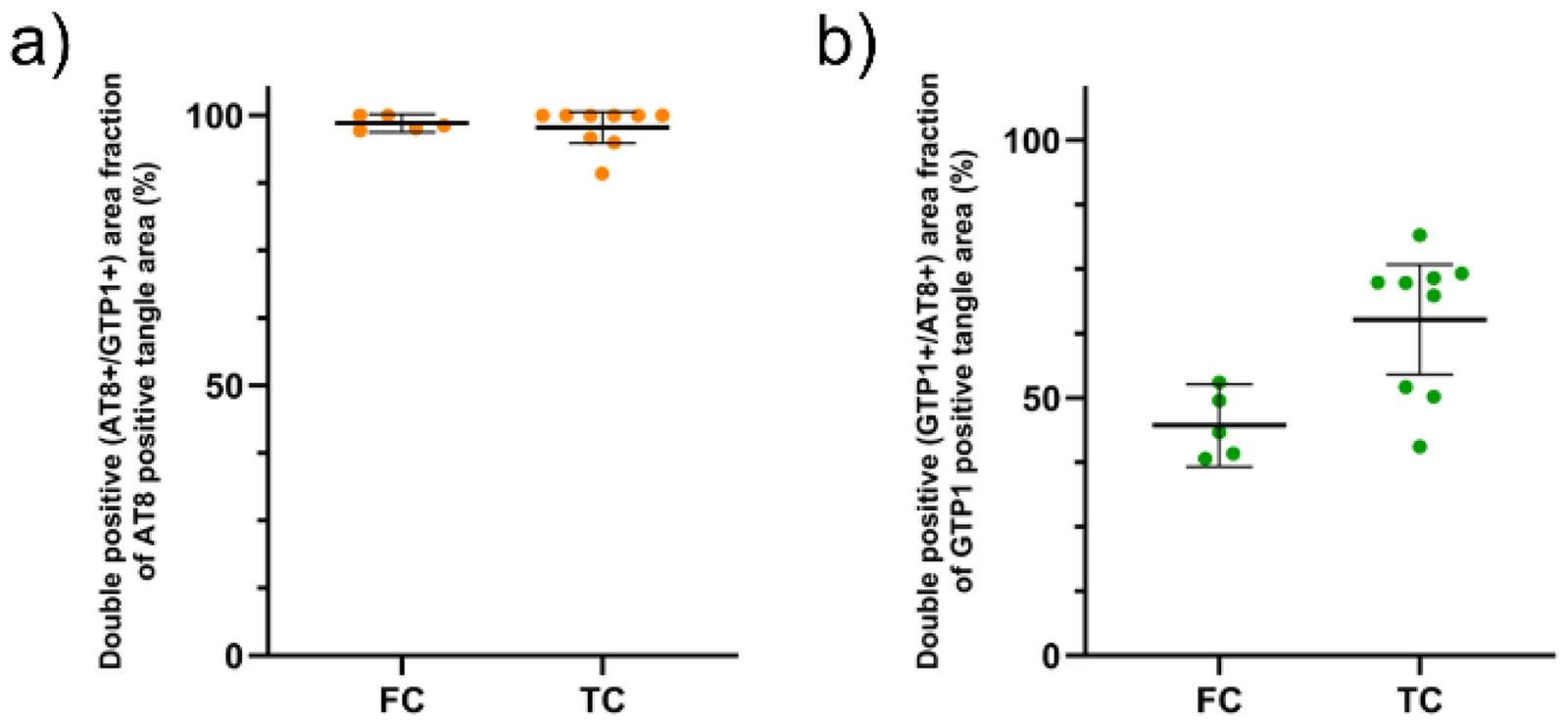
GTP1 stains AT8-positive tangles, but AT8 does not always stain GTP1-positive tangles a) The AT8-positive tangle area is almost entirely also GTP1-positive. The shown values represent area fraction occupied by double positive (AT8+/GTP1+) area within AT8-positive tangle area. b) GTP1-positive tangles are sometimes not captured by AT8. The shown values represent area fraction occupied by double positive (AT8+/GTP1+) area within AT8-positive tangle area. (a - b) Each data point corresponds to one patient (5 patients for FC and 9 patients for TC). The error bars represent 95% confidence interval.

**Figure 6:**
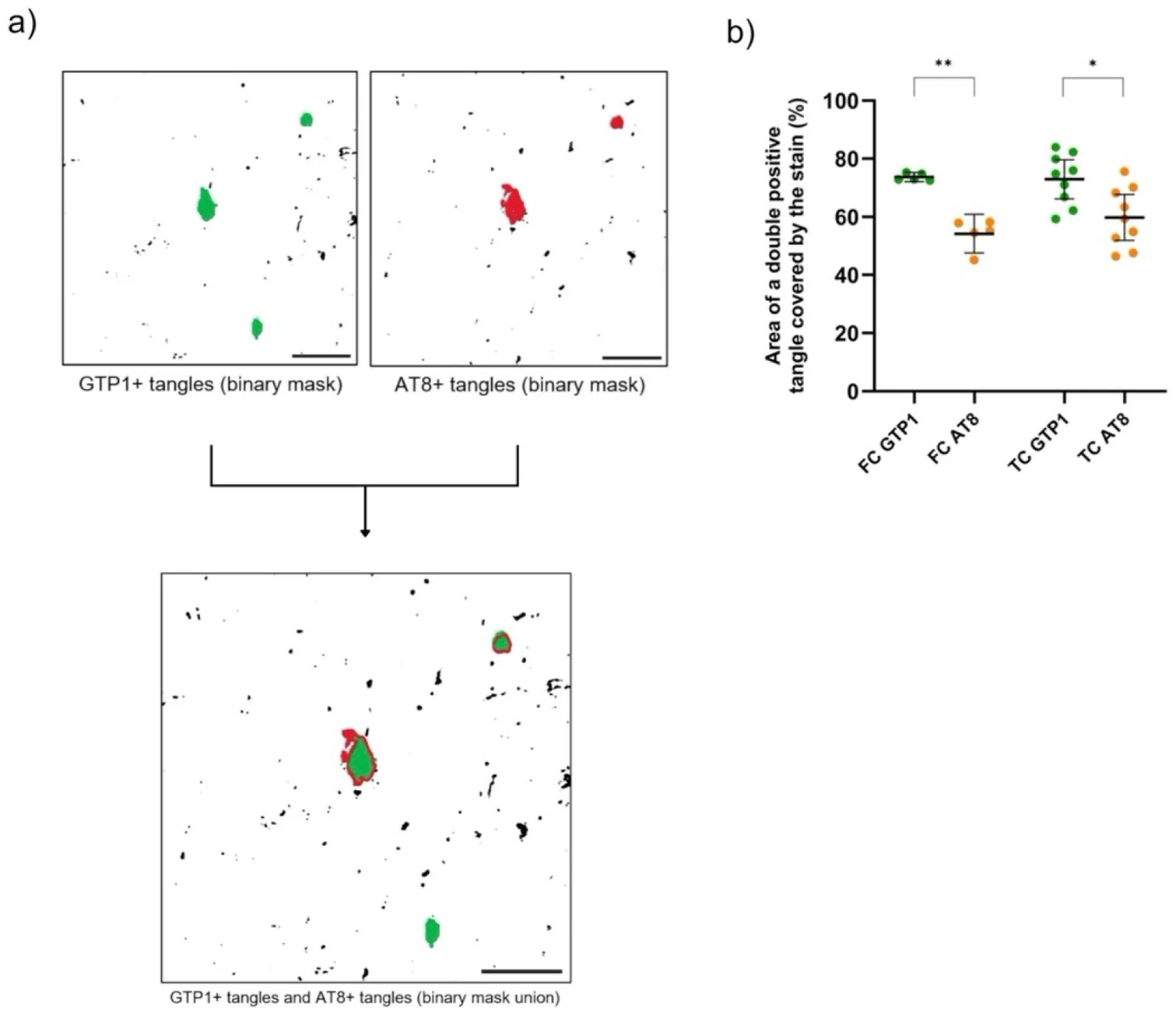
GTP1 stains a larger area within a single tangle compared to AT8 a) Method for determining the total area of individual tau tangles. Binary masks were generated from the FOV images of the GTP1 fluorescent signal and of the AT8 staining. Tau tangles ROIs were selected (described in the Methods section). The binary mask for GTP1 and the corresponding binary mask for AT8 were combined in a “union” image, where the ROIs of total tau tangles areas were selected and measured. b) The average area covered by GTP1 within a single tangle is significantly larger than that covered by AT8, both in frontal cortex (5 patients, paired t-test, p = 0.022, CI = [−27.25, −11.72]) and temporal cortex (9 patients, paired t-test, p = 0.033, CI = [−24.86, −1.381]). Area of a tangle covered by the stain is given as a fraction within a total area of a tangle (%). Each data point corresponds to one patient (5 patients for FC and 9 patients for TC). The error bars represent 95% confidence interval (CI). Scale bar: 50 μm.

**Table 1:** Demographic data for the included cases Alzheimer’s disease is the pathological diagnosis for all of the cases. Abbreviations: AAO – age at onset, AAD – age at death, FC – frontal cortex, TC – temporal cortex, M – male, F – female, SVD – small vessel disease, CAA – cerebral amyloid angiopathy, NA – not available.

Case	Sex	ABC score	Additional pathology	Clinical diagnosis	AAO	AAD	FC included	TC included
AD1	M	A3B3C3	SVD, TDP-43	Alzheimer’s disease	63	79	Yes	Yes
AD2	F	A3B3C3	-	Posterior cortical atrophy	55	68	Yes	No
AD3	M	A3B3C3	Cerebral infarction, CAA, Lewy bodies	Alzheimer’s disease	48	63	Yes	Yes
AD4	F	A3B3C3	-	Posterior cortical atrophy	57	65	Yes	No
AD5	F	A3B3C2	CAA, alpha-synuclein	Alzheimer’s disease	43	58	Yes	Yes
AD6	M	A3B3C3	CAA, SVD	Alzheimer’s disease	54	65	No	Yes
AD7	M	A3B3C3	Limbic Lewy body pathology, CAA	Posterior cortical atrophy	58	68	No	Yes
AD8	F	A3B3C3	CAA, SVD, amygdala Lewy body pathology, atheroma	Primary progressive aphasia	55	67	No	Yes
AD9	F	A3B3C2	NA	Alzheimer’s disease	49	69	No	Yes
AD10	F	A3B3C3	Hippocampal sclerosis	Amnestic disorder	50	66	No	Yes
AD11	F	A3B3C3	NA	Alzheimer’s disease	51	62	No	Yes
